# An Adult Patient with Fontan Physiology: A TEE Perspective

**DOI:** 10.1155/2012/475015

**Published:** 2011-12-08

**Authors:** Edward Gologorsky, Angela Gologorsky, Eliot Rosenkranz

**Affiliations:** ^1^Anesthesiology, Miller School of Medicine, Jackson Memorial Hospital, University of Miami, Miami, FL 33136, USA; ^2^Anesthesiology, Memorial Regional Hospital East, Hollywood, FL 33136, USA; ^3^Division of Cardiothoracic Surgery, Miller School of Medicine, Jackson Memorial Hospital, University of Miami, FL 33136, USA

## Abstract

Fontan and Baudet described in 1971 the separation of the pulmonary and systemic circulations resulting in univentricular physiology. The evolution of the Fontan procedure, most notably the substitution of right atrial-to-pulmonary artery anastomosis with cavopulmonary connections, resulted in significantly improved late outcomes. Many patients survive well into adulthood and are able to lead productive lives. While ideally under medical care at specialized centers for adult congenital cardiac pathology, these patients may present to the outside hospitals for emergency surgery, electrophysiologic interventions, and pregnancy. This presentation presents a “train of thought,” linking the TEE images to the perioperative physiologic considerations faced by an anesthesiologist caring for a patient with Fontan circulation in the perioperative settings. Relevant effects of mechanical ventilation on pulmonary vascular resistance, pulmonary blood flow and cardiac preload, presence of coagulopathy and thromboembolic potential, danger of abrupt changes of systemic vascular resistance and systemic venous return are discussed.

## 1. Introduction

Separation of pulmonary and systemic circulations, initially conceived as a palliation for tricuspid atresia but subsequently expanded to include other causes of univentricular physiology was described forty years ago, in 1971, by Fontan and Baudet. The evolution of the Fontan procedure, most notably the substitution of right atrial-to-pulmonary artery anastomosis with cavopulmonary connections, resulted in significantly improved late outcomes. As the result, many patients survive well into adulthood, fall under NY Heart Association (NYHA) functional classes I and II, and are able to lead productive lives [1]. Therefore, anesthesiologists may occasionally see these patients outside of specialized centers for adult congenital cardiac pathology; examples may include emergency surgery, electrophysiologic interventions, and pregnancy [2]. This presentation illustrates, from a TEE perspective, some of the unique challenges faced by an anesthesiologist caring for a patient with Fontan circulation in the perioperative settings. Consent for this presentation was obtained from the patient.

## 2. Case Description

A 31-year-old patient presented for replacement of a fractured epicardial lead and pacemaker pulse generator. His past surgical history was significant for a number of palliative interventions for a double-outlet right ventricle (DORV) with d-transposition of the great arteries and partial anomalous pulmonary venous return, culminating with a Fontan procedure, as well as a Maze procedure with epicardial pacemaker that leads placement for symptomatic atrial tachyarrhythmias. His medications included Warfarin for venous thromboembolism prophylaxis.

A preoperative conference with the surgeon allowed for a thorough anatomical and functional analysis. The prior Glenn and Fontan interventions resulted in total cavopulmonary connections: superior vena cava (SVC) to the right pulmonary artery and inferior vena cava (IVC) to the main pulmonary artery via a lateral conduit. Pulmonary arterial flow was wholly supported by the venous pressure from the great veins without any ventricular contribution. Therefore, adequate systemic venous pressures and maintenance of low pulmonary vascular resistance and alveolar pressures were paramount. Pulmonary venous return was divided between the left and right atria (the right pulmonary veins to the remnant of the right atrium, due to partially anomalous pulmonary venous return and the left pulmonary veins to the left atrium), which communicated with each other via an atrial septal defect (ASD). Right ventricle (RV) received right atrial (oxygenated) blood via tricuspid valve; left ventricle (LV) received the left atrial blood through the mitral valve. Both ventricles, communicating via a large nonrestrictive ventricular septal defect (VSD), contributed to the systemic cardiac output, since the aorta originated from the right ventricle (RV), and VSD directed the left ventricular outflow to aorta. The left atrial appendage was removed as part of the prior Maze procedure.

General anesthesia and transesophageal echocardiography (TEE) were requested for the planned reentry thoracotomy in this patient with mild symptomatic heart failure (NYHA functional class II). Having secured peripheral venous access and direct (radial) arterial pressure monitoring, general anesthesia was slowly induced with divided doses of midazolam and fentanyl. Once the pressure-limited positive pressure ventilation was judged to be tolerated hemodynamically, trachea was intubated with a single lumen tube, facilitated with vecuronium. The intervention was performed in right semilateral position. Single lung ventilation was not required.

TEE midesophageal four-chamber view (Supplementary Video clip 1 available online at doi: 10.1155/2012/475015) visualized the remnant of the right atrium (receiving right pulmonary venous return) communicating with the hypertrophied, hypocontractile right ventricle via the (competent) tricuspid valve. The left atrium (sans appendage) received the left pulmonary veins and communicated with the normally contracting left ventricle via a (competent) mitral valve. Large nonrestrictive ASD and VSD were identified.

A patent IVC-PA communication (lateral tunnel) was seen immediately to the right of the right atrial remnant (Video clip 1). Slight withdrawal of the probe allowed the examination of the IVC-PA connection (Video clip 2) and the visualization of severe intrahepatic dilation of IVC (Video clip 3). Spontaneous echo contrast in the IVC and in the IVC-PA communication suggested low-velocity flow; color Doppler interrogation of the anastomosis revealed a laminar pattern.

Forward rotation to midesophageal long-axis view (Video clip 4) allowed the examination of the RV conus and the left ventricular outflow through a large nonrestrictive VSD. The aorta was seen arising from the heavily trabeculated, hypertrophied, and dilated RV.

**Figure 1:**
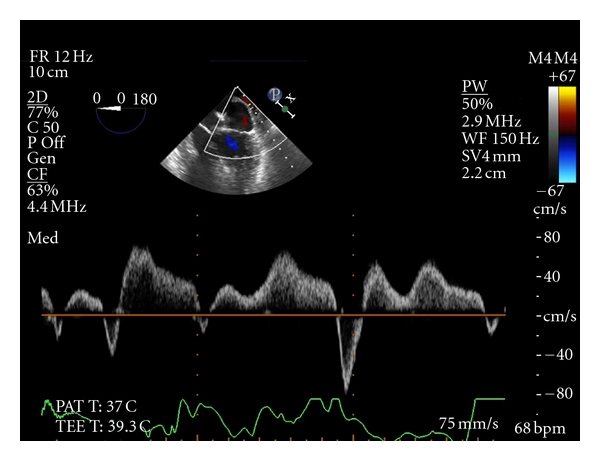
Pulse wave Doppler interrogation of the left superior pulmonary vein. Ar wave of 80 cm/sec is noted.

Deep transgastric long-axis view (Video clip 5) confirmed preserved contractility of the left ventricle and significantly hypertrophied right ventricle. Color Doppler examination of the left ventricular outflow towards the aorta via the nonrestrictive VSD revealed a laminar pattern (Video clip 6), without any significant atrioventricular regurgitation. Pulse wave Doppler interrogation of the left superior pulmonary vein revealed a deep (80 cm/sec) Ar wave, indicating significant diastolic dysfunction (Figure 1).

Intraoperatively, pressure-limited mechanical ventilation was tolerated very well. Upon the completion of the procedure intercostal nerves blocks were performed. Once adequate spontaneous ventilation was restored, the patient was extubated and returned to the intensive care unit for overnight observation. He was uneventfully discharged from the hospital two days later.

## 3. Discussion

**Figure 2:**
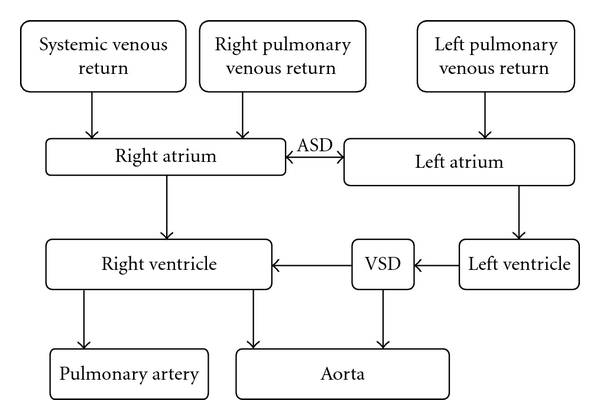
A schematic diagram of the blood flow in a patient with double-orifice right ventricle and partial anomalous pulmonary venous return prior to palliation. Right ventricle drives pulmonary and systemic circulations in parallel.

A schematic of the described patient's circulation at birth is presented in Figure 2. A double outlet right ventricle (DORV) is a type of ventriculoarterial connection in which both great arteries originate entirely or predominantly from the right ventricle; a large nonrestrictive VSD serves as the only left ventricular outlet [3]. Although in the majority of cases the aorta spirals posterior and obliquely to the pulmonary artery, in 30% it is found to course parallel and anterior to the pulmonary artery, resembling transposition of the great arteries. Therefore, the RV contributed to both the pulmonary and systemic circulation (making the patient's original pulmonary and systemic circuits functionally parallel). Extensive intracardiac mixing of oxygenated and deoxygenated blood at multiple levels (ASD, VSD, and partial anomalous pulmonary venous return) was essential for the patient's survival, but contributed to significant RV volume overload as well.

Partial anomalous pulmonary venous return describes the return of some pulmonary venous blood into the systemic venous (right) atrium site rather than the pulmonary venous (left) atrium. Typically, one or both right pulmonary veins fail to incorporate into the left atrium during embryogenesis and connect instead to the venae cavae or to the right atrium. In our patient, oxygenated blood from the right pulmonary veins mixes with the systemic blood return to the right atrium, and the left pulmonary veins return oxygenated blood via the left atrium into the left ventricle.

**Figure 3:**
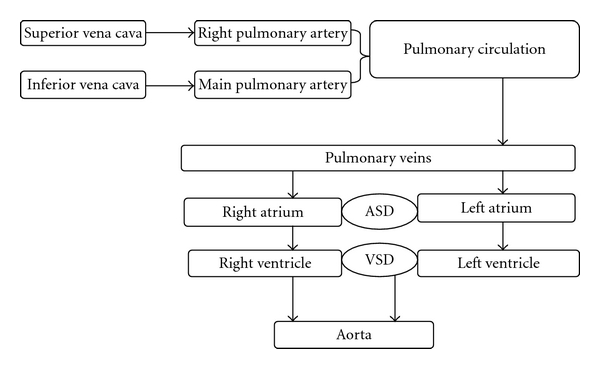
A schematic diagram of the blood flow in the presented patient after Fontan palliation. Pulmonary circulation is determined by the systemic venous return and the pulmonary vascular resistance.

A schematic of the described patient's circulation after the palliation is presented in Figure 3. The goal of surgical palliation was to decrease the demand on the RV by separating the pulmonary and systemic circuits and placing them in series rather than in parallel series. This arrangement-systemic venous return driving pulmonary artery circulation without a ventricular interposition-is the quintessential characteristic of Fontan circulation [4, 5].

The classical Fontan operation, consisting of right atriopulmonary connections, resulted in nonlaminar flow hydrodynamics (with consequential loss of the potential energy necessary to drive pulmonary artery flow), right atrial dilation, clot formation, and arrhythmias. Therefore, total cavopulmonary connections, omitting the right atrium, are preferred [6]. The goal of cavopulmonary connections is to maintain laminar blood flow as the patient grows. Our patient had first undergone a SVC-to-pulmonary artery connection (“bidirectional Glenn”), followed later with an IVC-to-pulmonary artery connection via a “lateral tunnel” (utilizing prosthetic baffle and a portion of the right atrial lateral wall). Alternatively, an extracardiac conduit between the IVC and pulmonary artery could be used as well.

As a result, the burden of the pulmonary circulation was removed from the RV. The absence of ventricular pump results in low velocity, nonpulsatile pulmonary blood flow, driven only by venous pressures, and critically dependent on low pulmonary vascular resistance [5, 7]. Pulmonary artery blood flow (and, therefore, cardiac output) variation is significantly related to the respiratory cycle [8], with marked augmentation during the inspiratory phase (in a spontaneously breathing patients) and profound decreases during the Valsalva maneuver. Hepatic blood flow augmentation appears to be the most significant contributor to increased pulmonary flow associated with spontaneous breathing [7]. Conversely, an inverse linear correlation was found between the mean airway pressures during positive pressure ventilation and the cardiac index, underlining a delicate balance between adequate mechanical ventilator support (aimed to prevent atelectasis formation, hypercarbia, and hypoxemia, all associated with increase in pulmonary flow resistance) and the cardiac performance of a patient with Fontan physiology [5].

**Figure 4:**
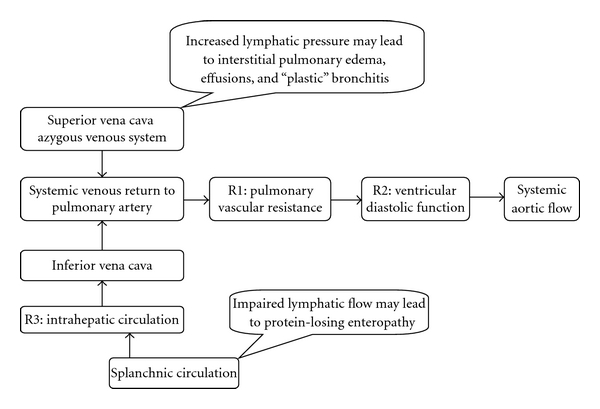
A schematic of resistors in series (R1, R2 and R3) imposed on the blood flow in Fontan palliation. Increase in pulmonary vascular resistance and deterioration of the ventricular compliance may result in decreased pulmonary and systemic flows.

Functionally, Fontan physiology imposes several resistors in series for blood return to the aortic circulation (Figure 4). In this patient, a large and hypertrophied RV is the main contributing force to the systemic circulation, while the output of the underloaded small LV reaches the aorta via the nonrestrictive VSD. Factors able to limit the systemic cardiac output include low preload, poor diastolic relaxation, usually associated with ventricular hypertrophy, and a high afterload. Sinus rhythm and low pulmonary vascular resistance are paramount to ventricular preload. The latter represents the main resistor to the systemic venous return to the ventricle. Part of the original Fontan's “ten commandments” for patient selection, pulmonary vascular resistance remains (along with the ventricular performance) the crucial factor affecting surgical outcomes [1]. Poor diastolic relaxation may further limit the ventricular preload and may be a predictor for short-term outcomes in Fontan patients [9] and serves as another resistor to the systemic flow. These same factors require aggressive attempts to maintain sinus rhythm, as tachyarrhythmias are particularly poorly tolerated in patients with Fontan circulation [4, 5]. Pressure increases upstream of venous resistors also account for complications such as decreased lymphatic drainage, protein-losing enteropathy, “plastic bronchitis,” pulmonary congestion, and pleural effusions [4, 5, 10].

An important consequence of chronically elevated central venous pressures, especially in the IVC basin, is the development of gradual hepatic congestion with attendant dysfunction and coagulopathy. Both pro and anticoagulant arms may be affected; reduced production of proteins C, S, and antithrombin III may predispose the sluggish venopulmonary blood flow to thrombus formation [4, 11]. Dehydration and infection may further increase the risk of a fatal pulmonary thromboembolism [4]. Covert chronic pulmonary microembolism (in up to 18% of the patients) may lead to pulmonary vascular occlusive disease and may require chronic anticoagulation [4, 11]. Spontaneous contrast formation is readily diagnosed by TEE and may be indicative of an increased risk for thromboembolism [8].

Therefore, the challenge of the intraoperative management of a patient with Fontan physiology is to maintain adequate perfusion pressure and cardiac output with minimal alterations in the pulmonary vascular resistance, cardiac rate and rhythm, and systemic venous blood return. Team approach and a thorough preoperative discussion with cardiologist and surgeon are paramount. Coagulopathy, iatrogenic, spontaneous, or mixed, frequently is a confounding factor. Risks and benefits of each anesthetic modality should be carefully weighed for each patient. Preservation of spontaneous ventilation and auxiliary effects of work of breathing on pulmonary blood flow, achieved with neuraxial, regional or local anesthetic, should be carefully balanced against the risks of coagulopathy and sudden changes in afterload and venous return. If general anesthesia is contemplated, untoward effects of positive pressure ventilation on systemic venous return and pulmonary hemodynamics should be carefully considered and minimized [12].

TEE provides an invaluable perioperative diagnostic and monitoring guidance, far superior to 2D transthoracic examination [8]. During the intraoperative echocardiographic examination, the midesophageal four-chamber view allows for the evaluation of the venopulmonary connections, the (bi) ventricular geometry, and performance. Low-velocity, laminar flow from the IVC towards the pulmonary artery, is examined for evidence of thrombi or obstruction. Because the pulmonary circulation is in series with the systemic circulation, such events would compromise the pulmonary circulation and may lead to decreases in ventricular preload, cardiac failure, and hypotension. The atria are examined for thrombus formation and function: sinus rhythm is especially important for the chronically underloaded ventricles.

Forward rotation of the transducer visualizes the ventricular outflow tracts and allows for further evaluation of the geometry and function of both ventricles. Ventricular hypertrophy and function, as well as the size and location of the VSD can all be visualized in this view. The absence of the fibrous continuity between the semilunar and atrioventricular valves (aortic and tricuspid in our patient) is characteristic of the RV origin of the aorta. Examination of the ventricles' geometry, performance, and outflow is facilitated by the deep transgastric long-axis view.

In conclusion, a perioperative TEE examination of an adult patient with Fontan physiology should provoke a “train of thought”, linking the images to the perioperative physiologic considerations. A thorough familiarity with the preceding corrective and palliative surgeries for the primary pathology is crucial for the correct echocardiographic interpretation.

## Supplementary Material

Loop 1. The midesophageal four chambers view. Arrow points to the IVC-PA communication. Spontaneous echo contrast suggests a low velocity blood flow. RA, right atrial remnant. LA, left atrium. RV, right ventricle. LV, left ventricle. VSD, ventricular septal defect.
Loop 2. The upper esophageal view. Color Doppler examination of IVC-to-PA communication suggests a laminar flow. IVC, inferior vena cava. PA, pulmonary artery.
Loop 3. Spontaneous echo contrast (arrow) in the severely dilated inferior vena cava (IVC) suggests a low velocity flow with a potential for thrombi formation.
Loop 4. The midesophageal long axis view. Aorta originates from the coarsely hypertrophied right ventricle (RV). A non-restrictive ventricular septal defect (VSD) allows the left ventricular (LV) outflow towards the aorta. LA, left atrium. IVS, interventricular septum.
Loop 5. Deep transgastric long axis view. Left ventricular (LV) contractility is preserved, right ventricle (RV) is hypertrophied. Aorta is seen arising from the RV. A non-restrictive ventricular septal defect (VSD) allows the left ventricular (LV) outflow towards the aorta.
Loop 6. The midesophageal long axis view. Color Doppler of the LV outflow suggests a laminar flow.
